# Antimicrobial use among adult inpatients at hospital sites within the Canadian Nosocomial Infection Surveillance Program: 2009 to 2016

**DOI:** 10.1186/s13756-020-0684-2

**Published:** 2020-02-13

**Authors:** Wallis Rudnick, Michelle Science, Daniel J. G. Thirion, Kahina Abdesselam, Kelly B. Choi, Linda Pelude, Kanchana Amaratunga, Jeannette L. Comeau, Bruce Dalton, Johan Delport, Rita Dhami, Joanne Embree, Yannick Émond, Gerald Evans, Charles Frenette, Susan Fryters, Greg German, Jennifer M. Grant, Jennifer Happe, Kevin Katz, Pamela Kibsey, Justin Kosar, Joanne M. Langley, Bonita E. Lee, Marie-Astrid Lefebvre, Jerome A. Leis, Allison McGeer, Heather L. Neville, Andrew Simor, Kathryn Slayter, Kathryn N. Suh, Alena Tse-Chang, Karl Weiss, John Conly, the Canadian Nosocomial Infection Surveillance Program

**Affiliations:** 1 Public Health Agency of Canada, 130 Colonnade Rd, Ottawa, ON K2E 7L9 Canada; 2 SickKids, 555 University Ave, Toronto, ON M5G 1X8 Canada; 3 Université de Montréal, 2900 Boulevard Edouard-Montpetit, Montréal, QC H3T 1J4 Canada; 4 McGill University Health Centre, 1001 Boulevard Décarie, Montréal, QC H4A 3J1 Canada; 5 The Ottawa Hospital, 501 Smyth Rd, Ottawa, ON K1H 8L6 Canada; 6 IWK Health Centre, 5980 University Ave, Halifax, NS B3K 6R8 Canada; 7 Dalhousie University, 6299 South St, Halifax, NS B3H 4R2 Canada; 8 Alberta Health Services, 1620 29 St NW, Calgary, AB T2N 4L7 Canada; 9 London Health Sciences Centre, 800 Commissioners Rd E, London, ON N6A 5W9 Canada; 10 University of Waterloo, 200 University Ave W, Waterloo, ON N2L 3G1 Canada; 11 University of Western Ontario, 1151 Richmond St, London, ON N6A 3K7 Canada; 12 University of Manitoba, Winnipeg, MB R3T 2N2 Canada; 13 Shared Health Manitoba, Winnipeg, MB R3T 2N2 Canada; 14 Children’s Hospital Winnipeg, 840 Sherbrook St, Winnipeg, MB R3E 0Z3 Canada; 15 Hôpital Maisonneuve-Rosemont, 5415 Boulevard de l’Assomption, Montréal, QC H1T 2M4 Canada; 16 Kingston General Hospital, 76 Stuart St, Kingston, ON K7L 2V7 Canada; 17 Alberta Health Services, 10240 Kingsway Avenue, Edmonton, AB T5H 3V9 Canada; 18 Health PEI, 16 Garfield St, Charlottetown, PEI C1A 6A5 Canada; 19 University of British Columbia, 2329 West Mall, Vancouver, BC V6T 1Z4 Canada; 20 Infection Prevention and Control Canada, Red Deer, AB T4N 6R2 Canada; 21 North York General Hospital, 4001 Leslie St, North York, ON M2K 1E1 Canada; 22 Royal Jubilee Hospital, 1952 Bay St, Victoria, BC V8R 1J8 Canada; 23 Saskatchewan Health Authority, Saskatoon, SK S7N 0W8 Canada; 24 Stollery Children’s Hospital, Edmonton, AB T6G 2B7 Canada; 25 University of Alberta, Edmonton, AB T6G 2R7 Canada; 26 Sunnybrook Research Institute, 2075 Bayview Ave, Toronto, ON M4N 3M5 Canada; 27 Sinai Health System, 600 University Ave, Toronto, ON M5G 1X5 Canada; 28 University of Toronto, 27 King’s College Cir, Toronto, ON M5S Canada; 29 Dalla Lana School of Public Health, University of Toronto, 155 College St, Toronto, ON M5T 3M7 Canada; 30 Nova Scotia Health Authority, 1276 South Park St, Halifax, NS B3H 2Y9 Canada; 31 Sunnybrook Health Sciences Centre, 2015 Bayview Ave, Toronto, ON M4N 3M5 Canada; 32 SMBD-Jewish General Hospital, 3755 Chemin de la Côte-Sainte-Catherine, Montréal, QC H3T 1E2 Canada; 33 University of Calgary, 3330 Hospital Dr NW, Calgary, AB T2N 4N1 Canada

**Keywords:** Antimicrobial use, Hospital, Surveillance

## Abstract

**Background:**

Antimicrobial resistance is a growing threat to the world’s ability to prevent and treat infections. Links between quantitative antibiotic use and the emergence of bacterial resistance are well documented. This study presents benchmark antimicrobial use (AMU) rates for inpatient adult populations in acute-care hospitals across Canada.

**Methods:**

In this retrospective surveillance study, acute-care adult hospitals participating in the Canadian Nosocomial Infection Surveillance Program (CNISP) submitted annual AMU data on all systemic antimicrobials from 2009 to 2016. Information specific to intensive care units (ICUs) and non-ICU wards were available for 2014–2016. Data were analyzed using defined daily doses (DDD) per 1000 patient days (DDD/1000pd).

**Results:**

Between 2009 and 2016, 16–18 CNISP adult hospitals participated each year and provided their AMU data (22 hospitals participated in ≥1 year of surveillance; 11 in all years). From 2009 to 2016, there was a significant reduction in use (12%) (from 654 to 573 DDD/1000pd, *p* = 0.03). Fluoroquinolones accounted for the majority of this decrease (47% reduction in combined oral and intravenous use, from 129 to 68 DDD/1000pd, *p* < 0.002). The top five antimicrobials used in 2016 were cefazolin (78 DDD/1000pd), piperacillin-tazobactam (53 DDD/1000pd), ceftriaxone (49 DDD/1000pd), vancomycin (combined oral and intravenous use was 44 DDD/1000pd; 7% of vancomycin use was oral), and ciprofloxacin (combined oral and intravenous use: 42 DDD/1000pd). Among the top 10 antimicrobials used in 2016, ciprofloxacin and metronidazole use decreased significantly between 2009 and 2016 by 46% (*p =* 0.002) and 26% (*p* = 0.002) respectively. Ceftriaxone (85% increase, *p* = 0.0008) and oral amoxicillin-clavulanate (140% increase, *p* < 0.0001) use increased significantly but contributed only a small component (8.6 and 5.0%, respectively) of overall use.

**Conclusions:**

This study represents the largest collection of dispensed antimicrobial use data among inpatients in Canada to date. Between 2009 and 2016, there was a significant 12% decrease in AMU, driven primarily by a 47% decrease in fluoroquinolone use. Modest absolute increases in parenteral ceftriaxone and oral amoxicillin-clavulanate use were noted but contributed a small amount of total AMU. Ongoing national surveillance is crucial for establishing benchmarks and antimicrobial stewardship guidelines.

## Background

Antimicrobial resistance (AMR) is a serious and growing worldwide threat to our ability to prevent and treat infections. Patients with infections caused by resistant bacteria are at higher risk of death and incur higher healthcare costs [1–5]. The link between quantitative antibiotic use and the subsequent emergence of bacterial resistance is well documented [6]. Antimicrobial stewardship, which aims to optimize the appropriate indication, selection, dosing, route, and duration of antimicrobial therapy, is an important component of reducing overall antibiotic use and has been shown to improve patient safety [7]. Effective antimicrobial stewardship and comprehensive infection prevention and control programs have potential to limit the emergence and spread of AMR [8–10].

Systematic monitoring of antimicrobial use (AMU) helps identify opportunities for interventions and enables evaluation of effectiveness of antimicrobial stewardship programs. The European Surveillance of Antimicrobial Consumption Network has demonstrated that monitoring antibiotic use is valuable in garnering political commitment for successful stewardship campaigns [11].

National data on AMU in Canadian hospitals are limited. Taylor et al. reported on the prevalence of AMU within a network of Canadian hospitals from 2002 and 2009 [12]. These data provide cross-sectional antimicrobial dispensing results from 28 and 44 hospitals, respectively. Data collected in the Canadian Drug Store and Hospital Purchases (CDH) Dataset (administered by IQVIA) captures the national quantity of antimicrobials purchased by the hospital sector (i.e., acute care, long-term care, government redistribution centers, and government facilities), but relies on proprietary projection methods and does not directly measure antimicrobial dispensation. To address these gaps and limitations, a working group within the Canadian Nosocomial Infection Surveillance Program (CNISP) developed a surveillance program for select acute-care secondary and tertiary hospitals across Canada with the following five aims: 1) estimate national and regional AMU in secondary and tertiary care hospitals; 2) provide AMU benchmarks; 3) estimate AMU by specific ward-type (including ICU and non-ICU wards; medical, surgical, combined, ICU and other ward types); 4) evaluate trends and patterns of AMR across Canadian hospitals; and 5) identify whether a correlation between CNISP AMU data and CNISP AMR and *Clostridiodes difficile* data can be established. In addition, AMU data will provide useful and relevant benchmarking information to stakeholders and the public in support of antimicrobial stewardship interventions in Canada.

## Methods

### Setting and participating sites

CNISP is a collaborative effort of the Canadian Hospital Epidemiology Committee (CHEC), a subcommittee of the Association of Medical Microbiologists, and Infectious Disease (AMMI) and the Public Health Agency of Canada (PHAC). As of July 2019, 74 sentinel hospitals from across 10 provinces and one territory participate in the CNISP network. The results presented here represent the 22 adult hospitals that participated in CNISP AMU surveillance from 2009 to 2016.

CNISP established a working group for antimicrobial use in 2007/08. A pilot study was conducted between 2009 and 2013 and the program transitioned into a routine surveillance program in 2014. AMU data were collected from 2009 to 2013 based on fiscal years and then from 2014 to 2016 based on calendar years (two hospitals provided 2014 data in fiscal years). In 2013, implementation of an antimicrobial stewardship program became a required organizational practice for accreditation for Canadian acute-care hospitals [13].

### Data variables and collection

#### Adult inpatients

Adult patients were defined as those ≥18 years of age or those patients on wards where the majority of patients are ≥18 years of age. Surveillance included admitted adults, including admissions in emergency departments, and excluded admissions in long-term care wards. Non-admitted patients in emergency departments were excluded. Participating sites provided corresponding inpatient-day denominators for each fiscal or calendar year as appropriate. Participating sites provided either the total hospital-level adult inpatient days or inpatient days broken down by ward category.

#### Antimicrobial use

Participating sites provided total dispensed adult inpatient hospital AMU separated by type of antimicrobial, administration route (parenteral (IV) and oral) and, since 2014, ward category (ICU and non-ICU wards). All systemic antibacterial use was included in the surveillance using Anatomical Therapeutic Chemical (ATC) codes: J01s, P01AB01 (metronidazole oral) and A07AA09 (vancomycin oral) (Additional file 1: Table S1) [14]. The corresponding year's World Health Organization (WHO) ATC/DDD value was used to convert the quantity of antimicrobial to defined daily doses (DDDs) [14]. The following antimicrobials were considered special cases and handled as outlined: for sulfamethoxazole and trimethoprim (co-trimoxazole, J01EE01), 1.6 g = 1 DDD based on Health Canada Drug Product Database [15]; for erythromycin (J01FA01), 1.0 g = 1 DDD and, for erythromycin ethylsuccinate, 2.0 g = 1 DDD. For benzylpenicillin (J01CE01) and benzathine benzylpenicillin (J01CE08), data received in million units (MU) was converted to grams (0.6 g = 1 MU) and then converted to DDDs using the WHO ATC/DDD value.

#### Data analysis

Data files from participating sites were centrally converted into a common platform for analysis. National and regional rates of AMU were calculated and standardized per 1000 inpatient days (pd): rates were calculated as (total DDDs / total pd) * 1000. Antimicrobials were grouped by classes and subclasses according to the annual WHO ATC/DDD Index [14] (Additional file 1: Table S1). Characteristics of participating hospitals were compared over time. AMU data were used to rank the top antimicrobial agents used individually, by class/subclass, by year, by institution bed size and by ward type. Patient-day weighted linear regression was used to test for linear temporal trends with year treated as an ordinal variable. Robust (heteroscedasticity-consistent) standard errors were used to account for repeated measures at the same hospital in the regression models and to calculate 95% confidence intervals (95%CI) around point estimates. *P*-values of ≤0.05 were considered statistically significant. To examine effects of changes in the group of participating hospital sites over time, secondary analysis included only sites that participated in all surveillance years. Linear regression models using square root, log and reciprocal transformations of the dependent variable (AMU) were explored in secondary analysis. All analyses were done using SAS (version 9.4) software.

## Results

### Participating sites

Between 2009 and 2016, between 16 and 18 CNISP adult hospitals per year provided AMU data, with representation from 6 sites in western Canada, 15 in central Canada (Ontario/Quebec), and 1 in eastern Canada (in total, 22 hospitals from 5 different provinces participated in at least 1 year of surveillance; 11 hospitals participated in all years). During the pilot study, between 2009 and 2013, there were 4 sites each year with ≤200 beds, 7 sites with 201–500 beds and 6 sites with ≥501 beds. Between 2014 and 2016, there were 2–3 sites each year with ≤200 beds, 6–9 sites with 201–500 beds and 7–8 sites with ≥501 beds. Participating site characteristics are summarized in Table 1. Data specific to ICU and non-ICU wards were only available from 2014 to 2016.

**Table 1:** Site characteristics for adult acute-care hospitals participating in CNISP AMU surveillance, 2009–2016

Variable	2009	2010	2011	2012	2013	2014	2015	2016
Hospital Sites (adult-only facilities)	17	17	17	17	17	16	17	18
Inpatient Days	2,248,729	2,296,710	2,430,114	2,432,819	2,436,568	2,528,205	2,891,489	2,967,559
Regions								
West	5	5	5	5	5	4	6	6
Central	12	12	12	12	12	11	10	11
East	0	0	0	0	0	1	1	1
Hospital Bed Size								
≥ 501 beds	6	6	6	6	6	7	8	7
201–500 beds	7	7	7	7	7	6	7	9
≤ 200 beds	4	4	4	4	4	3	2	2
Hospital Type								
Teaching	17	17	17	17	17	16	17	18
Community	0	0	0	0	0	0	0	0
Year Type for Data Collection								
Fiscal year	17	17	17	17	17	2	0	0
Calendar year	0	0	0	0	0	14	17	18
Intensive Care Units								
Number of Hospitals with Intensive Care Unit (ICU) Data Available			–			14	14	15
Inpatient days			–			130,124	138,660	146,051
Regions								
West			–			4	5	5
Central			–			9	8	9
East			–			1	1	1
Hospital Bed Size								
≥ 501 beds			–			6	7	6
201–500 beds			–			6	6	8
≤ 200 beds			–			2	1	1
Hospital Type								
Teaching			–			14	14	15
Community			–			0	0	0
Year Type for Data Collection								
Fiscal year			–			0	0	0
Calendar year			–			14	14	15

### Trends in antimicrobial use

**Fig. 1:**
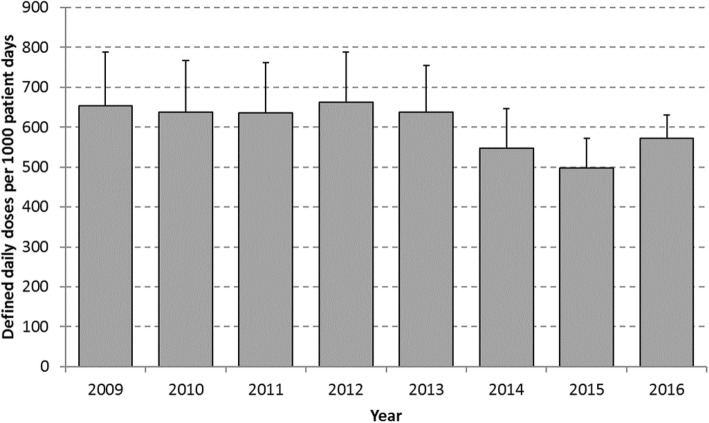
Total rate of antimicrobials used among adult inpatients at CNISP hospitals with 95% confidence intervals, 2009–2016

From 2009 to 2016 (Fig. 1), there was a significant decrease (12%, *p* = 0.02) in total AMU (from 654 (95%CI 519–789) to 573 (95%CI 514–631) DDD per 1000 patient days (/1000pd)). By hospital, the percentage change from 2009 to 2016 ranged from a 25% increase to a 52% decrease (median change in AMU at 13 hospitals with data available in 2009 and 2016: − 6%; interquartile range (IQR): − 19 to + 4%; decreases in AMU occurred at 8/13 hospitals). The five hospitals with an increase (0.7–25%) in antimicrobial use between 2009 and 2016 were all hospitals with relatively low AMU at the start (in 2009, 401–560 DDD/1000pd); AMU at three of the five hospitals remained below the median in 2016.

**Fig. 2:**
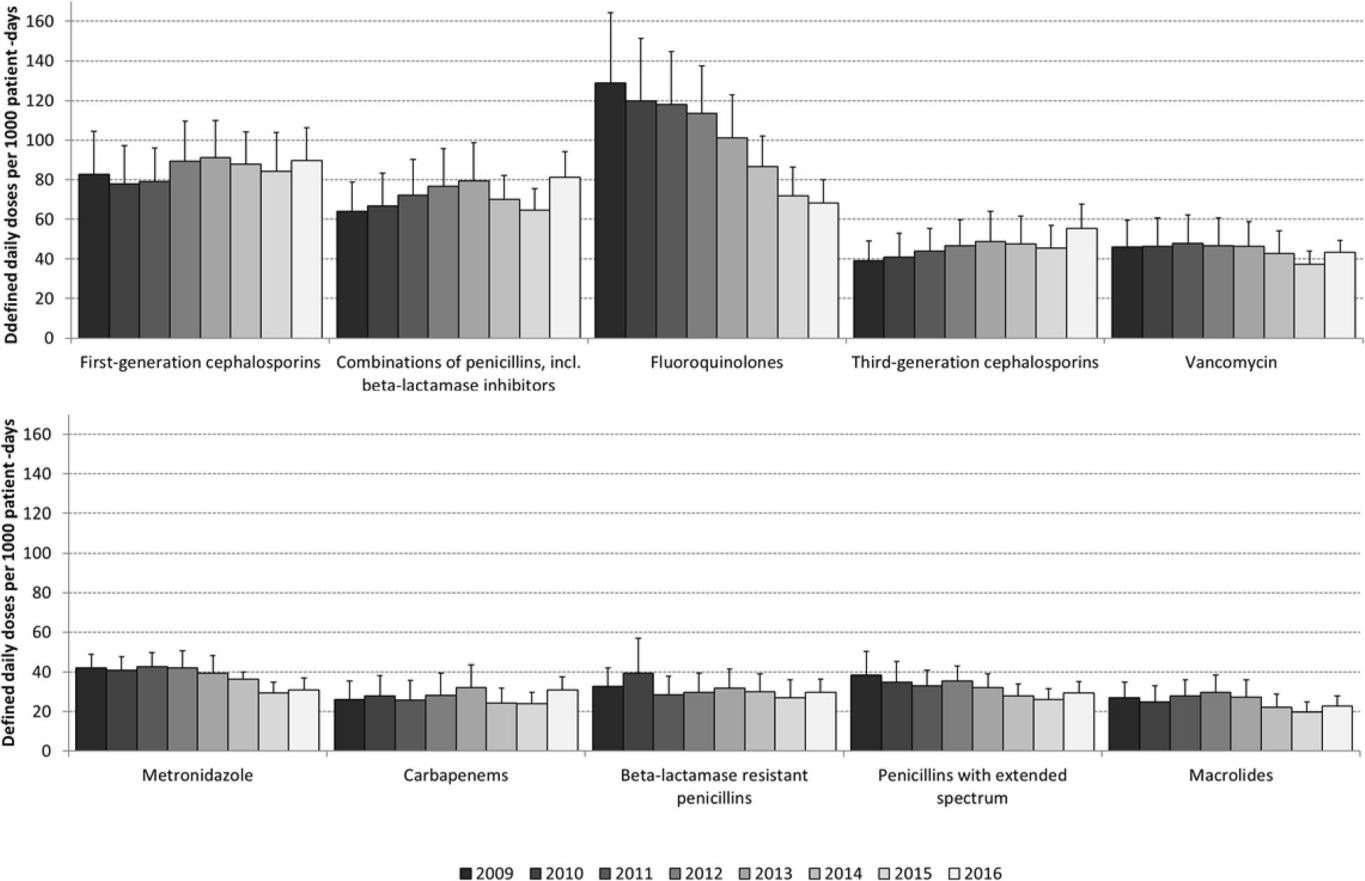
Total rate of antimicrobial classes/subclasses used among adult inpatients with 95% confidence intervals (2009–2016, top classes/subclasses in 2016)

**Fig. 3:**
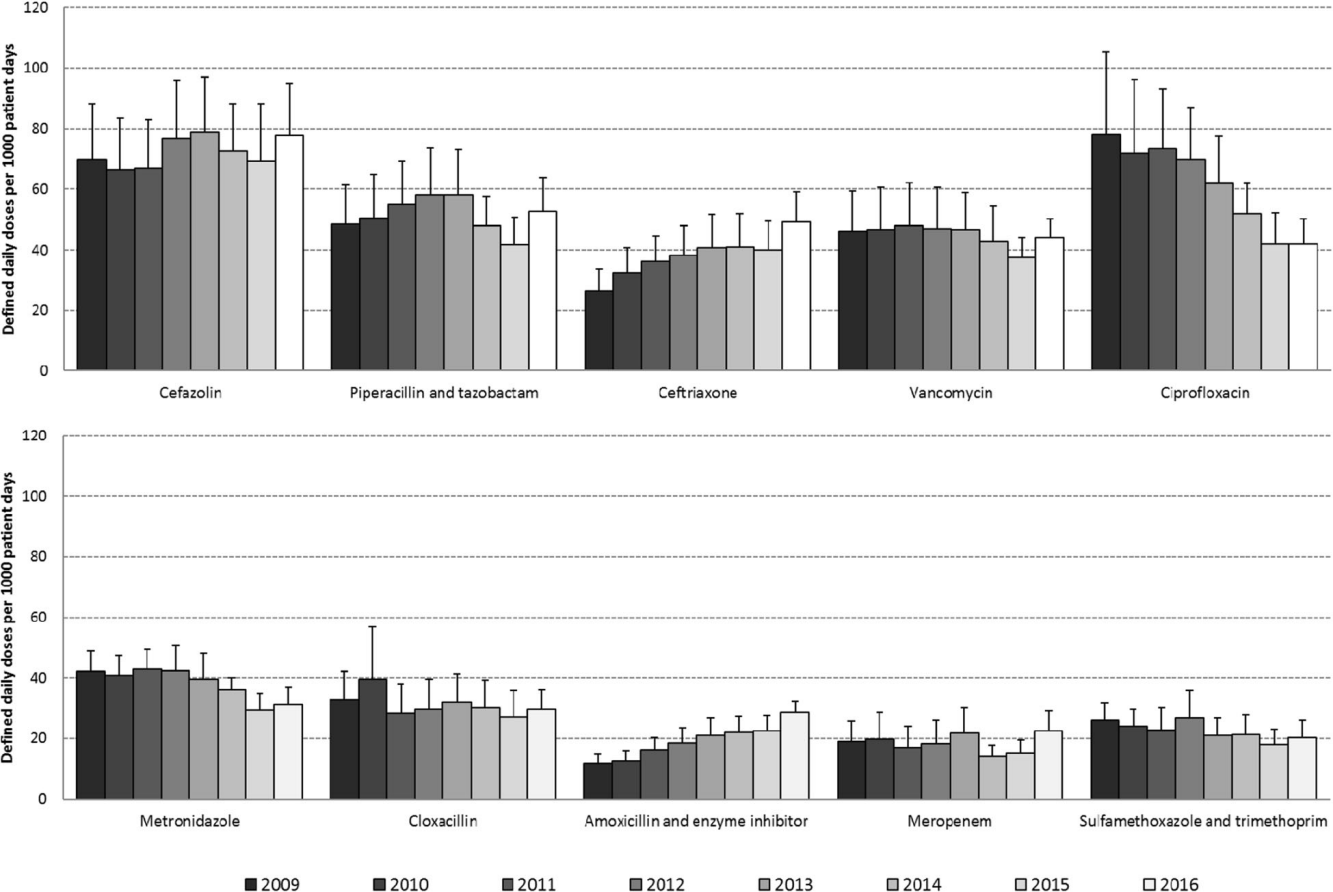
Total rate of antimicrobials used among adult inpatients with 95% confidence intervals (2009 to 2016, top antimicrobials in 2016)

The majority of the overall decrease in antimicrobial use was due to a 47% decrease in fluoroquinolone antibiotics (from 129 to 68 DDD/1000pd, *p =* 0.0002) (Fig. 2); use of each individual fluoroquinolone antibiotic decreased between 2009 and 2016 with ciprofloxacin decreasing from 78 to 42 DDD/1000pd (*p* = 0.002), levofloxacin decreasing from 38 to 20 DDD/1000pd (*p* = 0.02), and moxifloxacin decreasing from 11 to 6 DDD/1000pd (*p* = 0.04) (Fig. 3). Non-fluoroquinolone antibiotics decreased by only 4% (from 525 to 505 DDD/1000pd, *p* = 0.14). Decreases in fluoroquinolone use were seen at all hospitals (percentage decrease per hospital from 2009 to 2016 ranged from 13 to 80% for oral and IV use combined).

AMU varied substantially between hospitals but this variability decreased over time. In 2009, the IQR for overall AMU spanned 296 DDD/1000pd, while in 2016 the IQR spanned only 86 DDD/1000pd (IQR in 2009: 492–788 DDD/1000pd, IQR in 2016: 505–591 DDD/1000pd). This finding was due to large decreases in use among hospitals with high use in 2009 and smaller increases in use among hospitals with low use in 2009; among the five hospitals with the highest use in 2009, there was a median 30% decrease in use between 2009 and 2016 compared to a median 10% increase among the five hospitals with the lowest use in 2009 (for hospitals with data available in both 2009 and 2016). From 2009 to 2013 (Fig. 2), fluoroquinolones were the most frequently used class of agents and accounted for nearly 20% of all AMU in 2009. However, by 2014, fluoroquinolone use had decreased and first-generation cephalosporins became the most common class of agents used. In 2016, first generation cephalosporins represented 16% of all AMU (compared to 12% for fluoroquinolones). Of the top 10 antimicrobial classes/subclasses in 2016, use of fluoroquinolones (*p* = 0.0002), metronidazole (*p* = 0.002), penicillins with extended spectrum (i.e. amoxicillin, ampicillin, piperacillin and ticarcillin; *p* = 0.04) and macrolides (*p* = 0.03) decreased significantly between 2009 and 2016. Third-generation cephalosporins were the only class/subclass among the top ten in 2016 with significantly increased use between 2009 and 2016 (from 39 DDD/1000pd in 2009 to 55 DDD/1000pd in 2016, *p* = 0.02) but represented only 9.7% of antimicrobial use in 2016.

The top 10 antimicrobials used in 2016 (Fig. 3) were cefazolin (78 DDD/1000pd), piperacillin-tazobactam (53 DDD/1000pd), ceftriaxone (49 DDD/1000pd), vancomycin (oral and IV, 44 DDD/1000pd), ciprofloxacin (42 DDD/1000pd), metronidazole (31 DDD/1000pd), cloxacillin (30 DDD/1000pd), amoxicillin-clavulanate (i.e. amoxicillin and enzyme inhibitor; 29 DDD/1000pd), meropenem (23 DDD/1000pd) and trimethoprim-sulfamethoxazole (20 DDD/1000pd). Oral vancomycin (3 DDD/1000pd) represented 7% of vancomycin use in 2016 (data available for 15 hospitals).

Of the top 10 antimicrobials used in 2016, between 2009 and 2016, use of ciprofloxacin (46% decrease, *p =* 0.002) and metronidazole (26% decrease, *p* = 0.002) decreased significantly. Between 2009 and 2016, use of ceftriaxone (85% increase, *p* = 0.0008) and oral amoxicillin-clavulanate (140% increase, *p* < 0.0001) increased significantly but represented only a small component (8.6 and 5.0%, respectively) of overall use in 2016. Over the same period, cefazolin use increased from 70 to 78 DDD/1000pd (*p =* 0.59) and in 2012 became the single most frequently used antimicrobial. Clindamycin use represented 2% of total AMU in 2009, remained stable from 2009 to 2012 (~ 15 DDD/1000pd) and then decreased to 7 DDD/1000pd in 2016 *(p =* 0.004). Trends in use for specific antibiotics differed from one institution to another (data not shown). Secondary exploration of square root, log and reciprocal transformations of the dependent variable yielded no additional, significant non-linear associations.

**Fig. 4:**
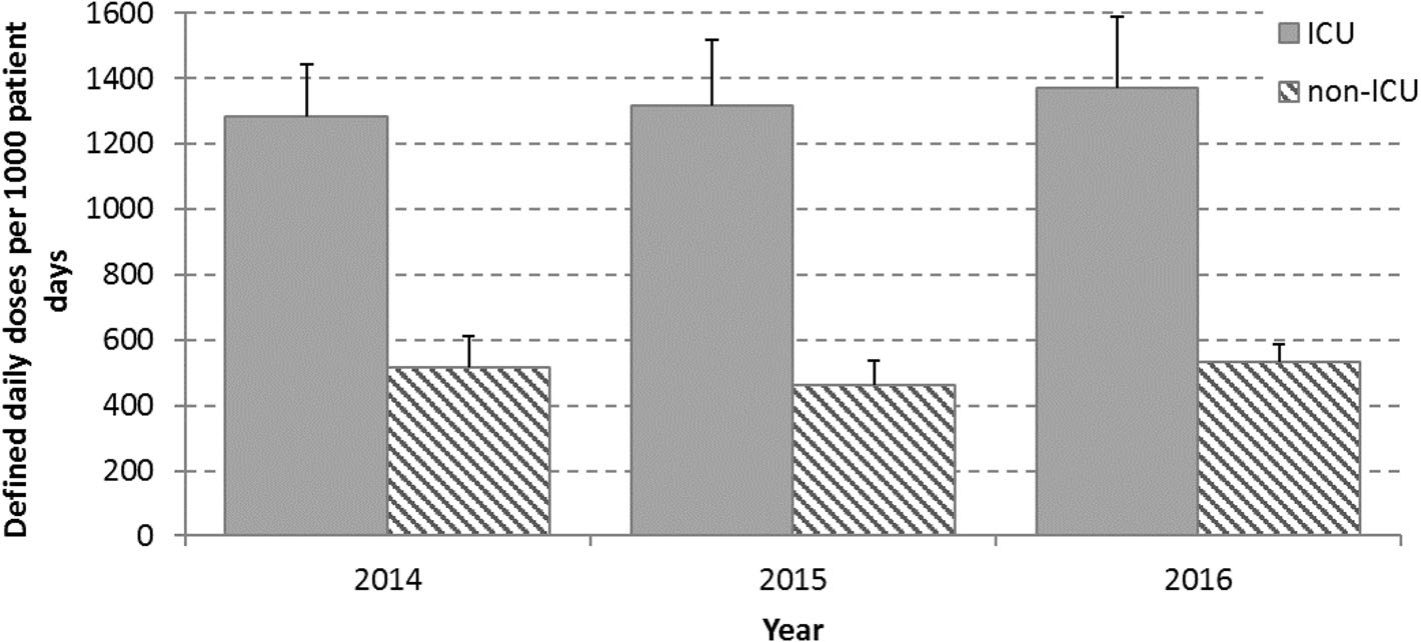
Total rate of antimicrobials used by adult inpatients by wardtype with 95% confidence intervals (*n* = 14–15 hospitals/year)

Although AMU among ICUs represented only a small proportion of the total AMU (12% of total DDDs in 2016), the rate of AMU was much higher in ICUs compared to non-ICU wards (Fig. 4). In 2016, overall AMU was 1373 DDD/1000pd on ICUs compared to 533 DDD/1000pd on non-ICU wards (the IQR for AMU on ICU wards was 1180 DDD/1000pd to 1470 DDD/1000pd). In ICUs in 2016, piperacillin-tazobactam (188 DDD/1000pd), vancomycin (oral and IV combined, 183 DDD/1000pd), cefazolin (152 DDD/1000pd), ceftriaxone (119 DDD/1000pd) and meropenem (118 DDD/1000pd) had the highest use. Among the top 20 antibiotics used in 2016, metronidazole was the only antibiotic used more commonly on non-ICU wards (23–29 DDD/1000pd on non-ICU wards vs 11–14 DDD/1000pd on ICU wards). Oral vancomycin was used more frequently in ICUs than in non-ICU wards (7 DDD/1000pd on ICUs vs 3 DDD/1000pd on non-ICU wards among the 15 hospitals with available data in 2016).

In secondary analysis of the 11 sites that participated in all years of surveillance, trends were consistent with the primary analysis; the overall rate of AMU decreased from 644 DDD/1000pd in 2009 to 561 DDD/1000pd in 2016 (vs. 654 to 573 DDD/1000pd using all participating hospitals; *p* = 0.04 for linear trend test among the 11 hospitals). No significant differences in AMU rates were found between bed size categories (*p* = 0.12, Additional file 1: Figure S1).

## Discussion

To date, these data represent the largest collection of dispensed antibiotic use data from adult hospitalized inpatients in Canada. Patterns and differences in AMU from 2009 to 2016 were identified. At participating CNISP acute-care hospitals (predominantly tertiary level), there has been an overall 12% decrease in total antibiotic use, largely due to a major reduction in the use of fluoroquinolone antibiotics. Over the same period, there was a corresponding, although proportionately smaller increase in cephalosporin use.

There have been changes in the types of antimicrobials used over time. The most notable changes were increases in the frequency of use of ceftriaxone (85%) and oral amoxicillin-clavulanate (140%); an almost 50% decrease in the use of ciprofloxacin; and, a more moderate (26%) decrease in the use of metronidazole. It is noteworthy that the increases in ceftriaxone and amoxicillin-clavulanate were only a small component of overall AMU and an increase in oral amoxicillin-clavulanate might suggest greater oral step-down therapy which is an important element of stewardship programs. The decrease in ciprofloxacin use was not offset by a concomitant increase in the rate of levofloxacin or moxifloxacin use. Clindamycin use decreased by 50% between 2012 and 2016.

It is unknown if the reduction in fluoroquinolone use is related to stewardship efforts or warnings of adverse effects associated with fluoroquinolone use in both the United States [16] and Canada [17] or a combination of both. Multiple warnings and safety communications regarding fluoroquinolone use were posted in the U.S. and Canada between 2008 and 2018 before, during and after the surveillance period. The U.S. Food and Drug Administration’s initial ‘black box’ warning in 2008 was related to tendinitis and tendon ruptures. Subsequent warnings included peripheral neuropathy (2013) and disabling side effects (2016). There were additional warnings posted after the surveillance period, in 2018, for “significant decreases in blood sugar and certain mental health effects” and “risk of ruptures or tears in the aorta” [16]. Changes in fluoroquinolone use may also be due to stewardship efforts, including improved prescribing, or increasing rates of resistance [18]. In 2007, the Infectious Diseases Society of America (IDSA) and the Society for Healthcare Epidemiology in America (SHEA) published guidelines on development of effective hospital-based antimicrobial stewardship programs [19]. In 2013, implementation of an antimicrobial stewardship program became a requirement of accreditation for Canadian acute-care hospitals [13]. Recent decreases in fluoroquinolone use are possibly not related to fears of causing *C. difficile* (CDI) as this association was known in the decade before the surveillance period; notably, in the early 2000s, there had been major hospital-associated CDI outbreaks in Quebec, Canada, that were strongly associated with fluoroquinolone use [20, 21].

Further study is needed to fully understand the effects of reductions in fluoroquinolone use in CNISP hospitals where there has been a concurrent reduction in the rates of hospital-associated MRSA and CDI. Notably, over the surveillance period, there was a reduction in CDI caused by strain type NAP1 (associated with ribotype 027 and highly fluoroquinolone resistant) at CNISP hospitals [22]. Reductions in CDI infections have been associated with decreased fluoroquinolone use in Canada [21], the United Kingdom [23], and the United States [24, 25]. In France, reductions in fluoroquinolone use have been found to be associated with a decrease in MRSA [26, 27] and fluoroquinolone-resistant *P. aeruginosa* rates [27]. In the U.S., reductions in fluoroquinolone use have been associated with decreases in the proportion of *S. aureus* and *E. coli* isolates that are MRSA or fluoroquinolone resistant, respectively [28].

Data on AMU are sparse and differences in measurements make comparisons difficult, but our rates are comparable with rates that have been reported elsewhere. A systematic review of international antibiotic consumption in acute care hospitals between 1997 and 2013 found that the pooled estimate of antibiotic consumption was 586 DDD/1000pd hospital-wide and was 1563 DDD/1000pd on ICU wards [29]. Our rates in 2014 hospital-wide (548 DDD/1000pd) and on ICU wards (1284 DDD/1000pd) are slightly less but within the same range. Our rate of hospital-wide use in 2014 is also similar to the rate reported for the same year from 17 teaching hospitals in Ontario, Canada, using purchasing data obtained from the company IMS Health (now IQVIA; 521 DDD/1000pd) [30].

AMU rates across hospitals within the same jurisdiction have been found to vary widely. Tan et al.’s study of Ontario hospitals found a 2.8-fold variability in overall AMU among 17 teaching hospitals [30]. Similarly among CNISP hospitals, there was variability seen between hospitals in overall rates of use as well as use by classes/subclasses (2.2-fold variability in overall use in 2016). Variations in AMU rates are likely related to differences in hospital services and specialties. Further study is needed to understand the drivers of this variability and determine how best to use benchmarks and stewardship programs in each setting.

Rates of AMU vary by ward type. Although rates of AMU are much higher on ICU wards compared to non-ICU wards, it is important to consider that interventions in the ICU will only have an impact on a small percentage of the total antibiotic use. Information on rates of AMU within specific hospital wards and within specific patient populations will help inform stewardship efforts. Oral vancomycin was used 2.3 times more frequently in ICUs compared to non-ICU wards; this is likely related to higher rates of severe CDI in ICUs [31].

Given that larger hospitals tend to have greater acuity and often multiple ICUs and specialized units caring for highly compromised patient populations, larger hospitals might be expected to have higher AMU than smaller institutions; however, Tan et al. found that, in Ontario, smaller hospitals had higher AMU [30] — potentially due to less developed stewardship activities. In our study, we did not see a major difference in the overall rate of AMU by hospital bed size; it is possible that we did not capture a representative dataset or that, despite variations in bed size, the patient acuity is similar given that hospitals participating in CNISP are generally larger, tertiary, urban acute-care teaching hospitals. Participating hospitals also may have more developed antibiotic stewardship programs than non-participating hospitals. Our sample size is also limited particularly for smaller hospitals and studies in smaller North American hospitals have found wide variability in AMU [30, 32].

We recognize that our study has limitations. The AMU data were collected only from teaching hospitals, were not collected from every province, and larger hospital sites were over represented. The data are at risk of selection bias related to hospitals opting to participate and maintain their participation in the surveillance project over time. We did not identify which hospitals had units (e.g., transplant units) or patient populations that would be expected to have higher levels of AMU. We also had no information on indication for use. There are known shortcomings to using DDDs to measure antibiotic exposure [33, 34]. The use of dispensed data may not represent what patients actually consume [35]. Although alterations of ATC and DDDs occur over time and it is recommended that past data be recalculated when these changes occur [36], it was not possible to recalculate DDD values. Linear regression models assume linearity and we did not test for non-monotonic trends.

## Conclusions

Our study describes 8 years of Canadian trends in hospital-based AMU and represents the largest collection of dispensed antibiotic use data from adult inpatients in Canada. There have been changes in the types of antimicrobials and antimicrobial classes used over time, notably a 47% decrease in fluoroquinolone use between 2009 and 2016 and an overall modest absolute increase in the use of ceftriaxone and amoxicillin-clavulanate. Over this same period, cefazolin use increased from 70 to 78 DDD/1000pd (*p =* 0.59) and, in 2012, became the single most frequently used antimicrobial agent. These results support the need for uniform, high-quality AMU and AMR surveillance to support ongoing stewardship efforts.

## Supplementary information

**Additional file 1: Figure S1.** Total rate of antimicrobials used among adult inpatients at CNISP hospitals, by hospital bed size category, 2009–2016. **Table S1.** ATC codes and defined daily doses (DDDs) for all systemic antibacterials included in CNISP AMU surveillance in 2016 (unless otherwise indicated DDD values are the 2016 World Health Organization ATC/DDD Index*).

## Data Availability

The aggregate national-level datasets used and/or analysed during the current study are available from the corresponding author on reasonable request. The hospital-level datasets generated and/or analysed during the current study are not publicly available due to the binding data sharing agreements with the hospitals involved in the surveillance program.
