# Supplementary material for: Antimicrobial use among adult inpatients at hospital sites within the Canadian Nosocomial Infection Surveillance Program: 2009 to 2016
Source: Antimicrob Resist Infect Control. 2020 Feb 13;9:32. doi: 10.1186/s13756-020-0684-2 (PMC7020554; doi:10.1186/s13756-020-0684-2)
Supplement: Supplementary file 1 — Additional file 1: Figure S1. Total rate of antimicrobials used among adult inpatients at CNISP hospitals, by hospital bed size category, 2009–2016. Table S1. ATC codes and defined daily doses (DDDs) for all systemic antibacterials included in CNISP AMU surveillance in 2016 (unless otherwise indicated DDD values are the 2016 World Health Organization ATC/DDD Index*). [file 13756_2020_684_MOESM1_ESM.doc]

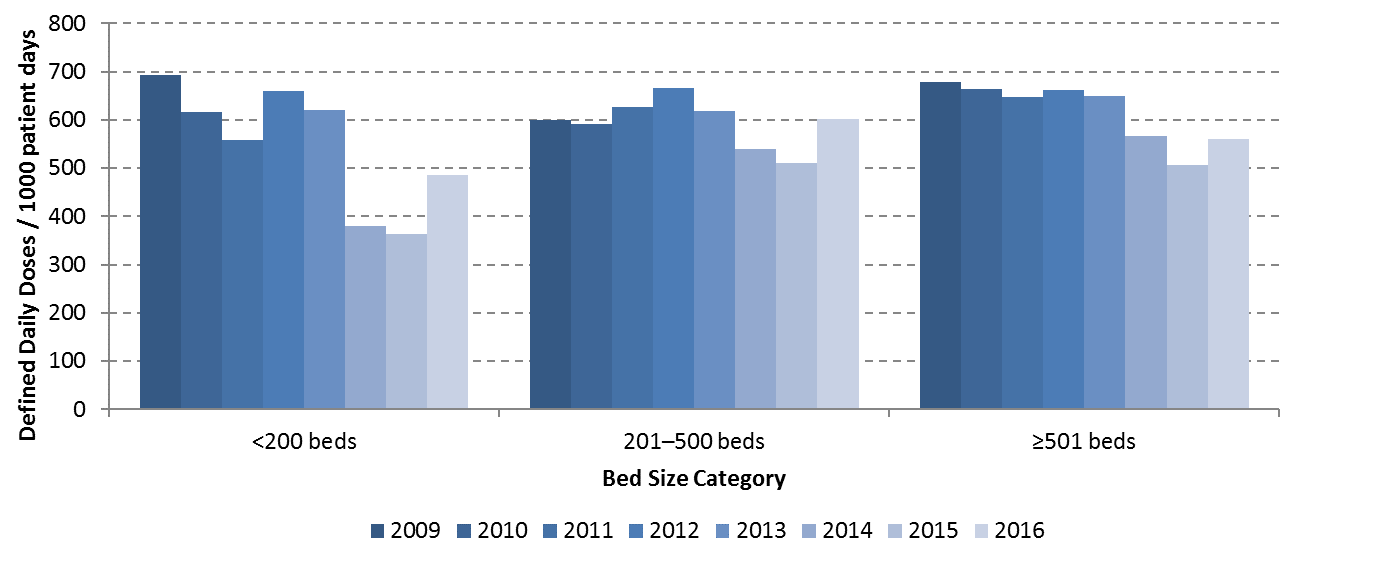


**Additional file 1**

**Figure S1.** Total rate of antimicrobials used among adult inpatients at CNISP hospitals, by hospital bed size category, 2009–2016

**Table S1.** ATC codes and defined daily doses (DDDs) for all systemic antibacterials included in CNISP AMU surveillance in 2016 (unless otherwise indicated DDD values are the 2016 World Health Organization ATC/DDD Index*)

| **ATC** | **Name** | **DDD** | **Unit** | **Route** | **Class as categorized** |
| --- | --- | --- | --- | --- | --- |
| J01BA01 | Chloramphenicol | 3 | g | Oral | Amphenicols |
| J01BA01 | Chloramphenicol | 3 | g | Parenteral | Amphenicols |
| J01CF02 | Cloxacillin | 2 | g | Oral | Beta-lactamase resistant penicillins |
| J01CF02 | Cloxacillin | 2 | g | Parenteral | Beta-lactamase resistant penicillins |
| J01CE08 | Benzathine benzylpenicillin | 3.6 | g | Parenteral | Beta-lactamase sensitive penicillins |
| J01CE01 | Benzylpenicillin (Penicillin G Sodium) | 3.6 | g | Parenteral | Beta-lactamase sensitive penicillins |
| J01CE02 | Phenoxymethylpenicillin (Penicillin V Potassium) | 2 | g | Oral | Beta-lactamase sensitive penicillins |
| J01DH04 | Doripenem | 1.5 | g | Parenteral | Carbapenems |
| J01DH03 | Ertapenem | 1 | g | Parenteral | Carbapenems |
| J01DH51 | Imipenem and cilastatin | 2 | g | Parenteral | Carbapenems |
| J01DH02 | Meropenem | 2 | g | Parenteral | Carbapenems |
| J01CR02 | Amoxicillin and beta-lactamase inhibitor | 1 | g | Oral | Combinations of penicillins, incl. beta-lactamase inhibitors |
| J01CR02 | Amoxicillin and beta-lactamase inhibitor | 1 | g | Parenteral | Combinations of penicillins, incl. beta-lactamase inhibitors |
| J01CR05 | Piperacillin and beta-lactamase inhibitor | 14 | g | Parenteral | Combinations of penicillins, incl. beta-lactamase inhibitors |
| J01CR03 | Ticarcillin and beta-lactamase inhibitor | 15 | g | Parenteral | Combinations of penicillins, incl. beta-lactamase inhibitors |
| J01EE01 | Sulfamethoxazole and trimethoprim^15^ | 1.6 | g | Oral | Combinations of sulfonamides and trimethoprim, incl. derivatives |
| J01EE01 | Sulfamethoxazole and trimethoprim^15^ | 1.6 | g | Parenteral | Combinations of sulfonamides and trimethoprim, incl. derivatives |
| J01DB05 | Cefadroxil | 2 | g | Oral | First-generation cephalosporins |
| J01DB01 | Cefalexin | 2 | g | Oral | First-generation cephalosporins |
| J01DB04 | Cefazolin | 3 | g | Parenteral | First-generation cephalosporins |
| J01MA02 | Ciprofloxacin | 0.5 | g | Oral | Fluoroquinolones |
| J01MA02 | Ciprofloxacin | 0.5 | g | Parenteral | Fluoroquinolones |
| J01MA12 | Levofloxacin | 0.5 | g | Oral | Fluoroquinolones |
| J01MA12 | Levofloxacin | 0.5 | g | Parenteral | Fluoroquinolones |
| J01MA14 | Moxifloxacin | 0.4 | g | Oral | Fluoroquinolones |
| J01MA14 | Moxifloxacin | 0.4 | g | Parenteral | Fluoroquinolones |
| J01MA06 | Norfloxacin | 0.8 | g | Oral | Fluoroquinolones |
| J01MA01 | Ofloxacin | 0.4 | g | Oral | Fluoroquinolones |
| J01MA01 | Ofloxacin | 0.4 | g | Parenteral | Fluoroquinolones |
| J01DE01 | Cefepime | 2 | g | Parenteral | Fourth-generation cephalosporins |
| A07AA09 | Vancomycin | 2 | g | Oral | Glycopeptide antibacterials (included under Antibiotics in ATC Index) |
| J01XA01 | Vancomycin | 2 | g | Parenteral | Glycopeptide antibacterials |
| J01XD01 | Metronidazole | 1.5 | g | Parenteral | Imidazole derivatives |
| J01EC02 | Sulfadiazine | 0.6 | g | Oral | Intermediate-acting sulfonamides |
| J01FF01 | Clindamycin | 1.2 | g | Oral | Lincosamides |
| J01FF01 | Clindamycin | 1.8 | g | Parenteral | Lincosamides |
| J01FF02 | Lincomycin | 1.8 | g | Oral | Lincosamides |
| J01FF02 | Lincomycin | 1.8 | g | Parenteral | Lincosamides |
| J01FA10 | Azithromycin | 0.3 | g | Oral | Macrolides |
| J01FA10 | Azithromycin | 0.5 | g | Parenteral | Macrolides |
| J01FA09 | Clarithromycin | 0.5 | g | Oral | Macrolides |
| J01FA09 | Clarithromycin | 1 | g | Parenteral | Macrolides |
| J01FA01 | Erythromycin | 1 | g | Oral | Macrolides |
| J01FA01 | Erythromycin | 1 | g | Parenteral | Macrolides |
| J01FA01 | Erythromycin ethylsuccinate tablets | 2 | g | Oral | Macrolides |
| J01DF01 | Aztreonam | 4 | g | Parenteral | Monobactams |
| J01XE01 | Nitrofurantoin | 0.2 | g | Oral | Nitrofuran derivatives |
| P01AB01 | Metronidazole | 2 | g | Oral | Nitroimidazole derivatives |
| J01XD01 | Metronidazole | 1.5 | g | Parenteral | Nitroimidazole derivatives (included under Imidazole derivatives in ATC Index) |
| J01GB06 | Amikacin | 1 | g | Parenteral | Other aminoglycosides |
| J01GB03 | Gentamicin | 0.24 | g | Parenteral | Other aminoglycosides |
| J01GB05 | Neomycin | 1 | g | Oral | Other aminoglycosides |
| J01GB01 | Tobramycin | 0.24 | g | Parenteral | Other aminoglycosides |
| J01XX09 | Daptomycin | 0.28 | g | Parenteral | Other antibacterials |
| J01XX01 | Fosfomycin | 3 | g | Oral | Other antibacterials |
| J01XX01 | Fosfomycin | 8 | g | Parenteral | Other antibacterials |
| J01XX08 | Linezolid | 1.2 | g | Oral | Other antibacterials |
| J01XX08 | Linezolid | 1.2 | g | Parenteral | Other antibacterials |
| J01XX05 | Methenamine hippurate | 2 | g | Oral | Other antibacterials |
| J01XX05 | Methenamine mandelate | 3 | g | Oral | Other antibacterials |
| J01DI01 | Ceftobiprole medocaril | 1.5 | g | Parenteral | Other cephalosporins and penems |
| J01CA04 | Amoxicillin | 1 | g | Oral | Penicillins with extended spectrum |
| J01CA04 | Amoxicillin | 1 | g | Parenteral | Penicillins with extended spectrum |
| J01CA01 | Ampicillin | 2 | g | Oral | Penicillins with extended spectrum |
| J01CA01 | Ampicillin | 2 | g | Parenteral | Penicillins with extended spectrum |
| J01CA12 | Piperacillin | 14 | g | Parenteral | Penicillins with extended spectrum |
| J01CA13 | Ticarcillin | 15 | g | Parenteral | Penicillins with extended spectrum |
| J01XB01 | Colistin | 3 | MU | Parenteral | Polymyxins |
| J01DC04 | Cefaclor | 1 | g | Oral | Second-generation cephalosporins |
| J01DC05 | Cefotetan | 4 | g | Parenteral | Second-generation cephalosporins |
| J01DC01 | Cefoxitin | 6 | g | Parenteral | Second-generation cephalosporins |
| J01DC10 | Cefprozil | 1 | g | Oral | Second-generation cephalosporins |
| J01DC02 | Cefuroxime | 0.5 | g | Oral | Second-generation cephalosporins |
| J01DC02 | Cefuroxime | 3 | g | Parenteral | Second-generation cephalosporins |
| J01XC01 | Fusidic acid | 1.5 | g | Oral | Steroid antibacterials |
| J01XC01 | Fusidic acid | 1.5 | g | Parenteral | Steroid antibacterials |
| J01FG02 | Quinupristin/dalfopristin | 1.5 | g | Parenteral | Streptogramins |
| J01GA01 | Streptomycin | 1 | g | Parenteral | Streptomycins |
| J01AA01 | Demeclocycline | 0.6 | g | Oral | Tetracyclines |
| J01AA02 | Doxycycline | 0.1 | g | Oral | Tetracyclines |
| J01AA02 | Doxycycline | 0.1 | g | Parenteral | Tetracyclines |
| J01AA08 | Minocycline | 0.2 | g | Oral | Tetracyclines |
| J01AA08 | Minocycline | 0.2 | g | Parenteral | Tetracyclines |
| J01AA07 | Tetracycline | 1 | g | Oral | Tetracyclines |
| J01AA07 | Tetracycline | 1 | g | Parenteral | Tetracyclines |
| J01AA12 | Tigecycline | 0.1 | g | Parenteral | Tetracyclines |
| J01DD08 | Cefixime | 0.4 | g | Oral | Third-generation cephalosporins |
| J01DD01 | Cefotaxime | 4 | g | Parenteral | Third-generation cephalosporins |
| J01DD02 | Ceftazidime | 4 | g | Parenteral | Third-generation cephalosporins |
| J01DD04 | Ceftriaxone | 2 | g | Parenteral | Third-generation cephalosporins |
| J01EA01 | Trimethoprim | 0.4 | g | Oral | Trimethoprim and derivatives |
| J01EA01 | Trimethoprim | 0.4 | g | Parenteral | Trimethoprim and derivatives |

* WHO Collaborating Centre for Drug Statistics Methodology. ATC/DDD Index. Available at: <https://www.whocc.no/atc_ddd_index/>^. Accessed June 16, 2019^.
